# Congenital anomalies after first-trimester dydrogesterone therapy during in vitro fertilization

**DOI:** 10.1186/s12958-025-01507-8

**Published:** 2025-12-05

**Authors:** Wan Yang, Lin Zeng, Lixue Chen, Rui Yang, Haiyan Wang, Ping Liu, Ying Lian, Rong Li, Hongbin Chi, Jie Qiao

**Affiliations:** 1 Center for Reproductive Medicine, Department of Obstetrics and Gynecology, Peking University Third Hospital, 49 N Garden Rd, Haidian District, Beijing, 100191 China; 2 National Clinical Research Center for Obstetrics and Gynecology, Key Laboratory of Assisted Reproduction (Peking University), Ministry of Education, Beijing Key Laboratory of Reproductive Endocrinology and Assisted Reproductive Technology, Beijing, 100191 China; 3 Research Centre of Clinical Epidemiology, Peking University Third Hospital, Beijing, China

**Keywords:** Congenital anomaly, Dydrogesterone, In vitro fertilization, Luteal phase support

## Abstract

**Background:**

Congenital anomalies are a critical public health concern and warrant prioritization in research. However, the teratogenic potential of dydrogesterone (DYG) remains uncertain and a subject of ongoing debate.

**Methods:**

This retrospective cohort study included patients undergoing embryo transfer between January 2010 and December 2018. It analyzed 124,815 embryo transfer cycles (80,103 [64.2%] fresh; 44,712 [35.8%] frozen), resulting in 52,175 live births. Newborns were stratified by maternal luteal phase support (DYG-exposed vs. unexposed). Patients with known congenital malformation risk factors were excluded. Congenital anomaly incidence was compared between groups. Stratified analysis and multivariate logistic regression models adjusting for confounders were employed.

**Results:**

The total congenital anomaly rate was significantly lower in DYG-exposed newborns compared to unexposed groups (6.05‰ vs. 7.90‰, *P* = 0.020), with particularly notable differences in musculoskeletal malformations (0.63‰ vs. 1.33‰, *P* = 0.025). No significant differences were observed in other congenital anomaly categories. After stratifying by fresh or frozen cycles, DYG-exposed and unexposed groups showed no significant differences in birth defects during fresh cycles. In frozen cycles, musculoskeletal anomalies were significantly lower in the DYG group both before (0.60‰ vs. 2.37‰, *P* = 0.020) and after adjustment (*P* = 0.009, OR: 0.19, 95% CI: 0.05–0.66). Other anomaly categories remained unaffected. However, this specific association did not remain significant after rigorous correction for multiple testing with the application of both the Bonferroni and False Discovery Rate (FDR) methods. Multivariate analysis adjusted for confounders revealed no increased risk of congenital anomalies associated with DYG exposure.

**Conclusions:**

First-trimester dydrogesterone therapy was not associated with an increased risk of congenital anomalies. Due to the limitations of this cohort study, further follow-up and in-depth data analysis are planned for future studies.

**Supplementary Information:**

The online version contains supplementary material available at 10.1186/s12958-025-01507-8.

## Introduction

Luteal phase defects (LPD) are strongly linked to controlled ovarian stimulation in in vitro fertilization (IVF) cycles [1, 2]. Proposed mechanisms include granulosa cell loss during oocyte retrieval, impairing progesterone synthesis, and GnRH agonist-induced suppression of luteinizing hormone (LH), which disrupts corpus luteum development [2, 3]. Consequently, luteal phase support (LPS) – a critical component of IVF protocols – is widely implemented to improve live birth rates [3].

Current luteal phase support (LPS) protocols utilize three progesterone formulations: vaginal, intramuscular (IM), and oral. Vaginal administration – employed in 64% of cycles [4] – achieves the highest endometrial progesterone concentrations due to uterine first-pass effects and avoidance of hepatic metabolism [1, 5], yet is associated with adverse effects like irritation, discharge, and bleeding [6]. In contrast, IM administration produces peak serum progesterone levels [5] but carries risks of injection-site pain, infections, and abscesses, often reducing patient compliance [1]. Oral formulations (e.g., micronized progesterone) undergo extensive pre-hepatic and hepatic metabolism, resulting in minimal uterine bioavailability [1, 5], and are clinically inferior to vaginal or IM routes in efficacy outcomes.

To address these limitations, dydrogesterone (DYG) –a retroprogesterone with good oral bioavailability – has been adopted for luteal phase support (LPS) in IVF [7]. DYG and its active metabolite, 20α-dihydrodydrogesterone, exhibit high progesterone receptor selectivity with almost no affinity for androgenic, estrogenic, or glucocorticoid receptors, suggesting considerable safety during pregnancy [8–10]. While randomized trials demonstrate comparable efficacy and safety between DYG and vaginal progesterone [6, 11–14], conflicting evidence exists: a case–control study reported elevated congenital anomaly risks with DYG exposure [15], and a review of 28 maternal DYG cases noted diverse fetal defects without establishing causality [16]. Other recent divergent findings include a Chinese prospective cohort linking first-trimester DYG to higher anomaly rates [17] versus WHO database analyses implicating DYG in hypospadias and cardiac defects relative to progesterone [18]. Given DYG’s widespread use in infertility management and our center’s annual IVF volume (> 17,000 cycles), this retrospective cohort study aimed to clarify birth defect risks following first-trimester DYG exposure in IVF pregnancies.

## Materials and methods

### Study design and population

This retrospective cohort study included patients undergoing embryo transfer (fresh or frozen cycles) between January 2010 and December 2018 at our hospital’s Reproductive Medicine Center. Participants were stratified into DYG-exposed and unexposed groups. The DYG-exposed group comprised patients receiving DYG for LPS, whether combined with intramuscular or vaginal progesterone. The unexposed group included those administered progesterone-only LPS without DYG. Exclusion criteria: preexisting endocrine/metabolic disorders such as diabetes mellitus, hypertension, thyroid dysfunction, and autoimmune disease; family history of birth defects, such as chromosomal abnormalities or congenital heart disease; recurrent miscarriage/implantation failure, considering the use of specific medications that could potentially be associated with neonatal congenital defects during IVF; and history of miscarriage, which is a risk factor for congenital malformations [19]. Analyses were stratified by fresh/frozen cycles due to protocol differences. Only live-born infants were included to assess societal burden relevance. The reproductive health ethics committee of Peking University Third Hospital approved this retrospective cohort study. Informed consent was waived due to the retrospective nature of the study (ethics approval document number IRB00006761-M2019487). Procedures performed in this study involving human participants or human tissue were in accordance with the ethical standards of the institutional and/or national research committee, and with the 1975 Helsinki Declaration and its later amendments or comparable ethical standards.

### Fresh embryo transfer and LPS

Oocytes were fertilized via conventional IVF or intracytoplasmic sperm injection (ICSI) based on semen parameters. LPS was initiated on oocyte retrieval day and continued until 10 weeks of gestation for confirmed viable pregnancies. Fresh cycle LPS protocols included: Oral dydrogesterone (DYG; Abbott Biologicals B.V.) 20 mg twice daily + intramuscular progesterone in oil (Shanghai Pharmaceutical) 20 mg daily; Vaginal progesterone gel 8% (Crinone®; Merck Serono) 90 mg daily; Intramuscular progesterone 60 mg daily (Table 1).

**Table 1:** DYG status and protocols among women who underwent fresh and frozen cycles

	DYG-exposed		DYG-unexposed	
	Number	protocols	Number	protocols
Fresh Cycles	1366	① DYG 20 mg BID + IM progesterone 20 mg QD;	78737	① IM progesterone 60 mg QD; ② Vaginal progesterone 90 mg QD;
Frozen natural cycles or stimulated cycles	29204	① DYG 10–20 mg BID;	1650	① IM progesterone 20–40 mg QD; ② oral micronized progesterone 200 mg QD;
Frozen replacement cycles	9901	① DYG 20 mg BID + IM progesterone 40 mg QD; ② DYG 20 mg BID + Vaginal progesterone 90 mg QD;	3957	① IM progesterone 60–80 mg QD;

*DYG* dydrogesterone, *LPS* luteal phase surpport, *BID* twice daily, *QD* once daily, *IM* intramuscular

### Frozen embryo transfer and LPS

Endometrial preparation for frozen embryo transfer (FET) varied across three protocols. Natural cycles were applied to patients with regular menses, initiating LPS three days post-ovulation. For irregular cycles, ovulation induction involved clomiphene, letrozole, or highly purified human menopausal gonadotropin (hMG, 75 IU/d; Livzon) until dominant follicles exceeded 18 mm, followed by hCG trigger (10,000 IU; Livzon). Natural/mild stimulation cycles utilized LPS with oral dydrogesterone 10–20 mg BID, intramuscular progesterone 20–40 mg daily, or oral micronized progesterone 200 mg daily (Utrogestan). Artificial cycles for thin endometrium history required estradiol valerate 2–3 mg BID (Progynova) from cycle days 2–3, with LPS starting post-endometrial thickness > 8 mm and ≥ 10 days of estrogen exposure. Artificial cycle LPS combined oral DYG 20 mg BID with intramuscular progesterone 40 mg daily or vaginal gel 90 mg daily, or intramuscular progesterone 60–80 mg daily (Table 1).

### Outcomes and definitions

The primary outcome was the incidence of congenital anomalies in live-born infants. Congenital anomalies, including minor and major, were defined as structural or functional anomalies occurring during intrauterine life. Non-invasive prenatal diagnosis using DNA technology or prenatal screening for Down’s syndrome was performed between 12 and 20^+6^ weeks of gestation. To screen for fetal anomalies, ultrasonography was scheduled between 18 and 24 weeks of gestation. Amniocentesis or fetal blood sampling was performed if any anomalies were detected using the prenatal screening methods described above. All live newborns underwent physical examinations at birth by a neonatologist to detect the presence of gross congenital anomalies. For newborn follow-up, trained nurses carried out telephone surveys on the 12^th^, 28^th^, and 37^th^ weeks of gestation and one month postpartum. All data was carefully and precisely recorded in the medical record system.

### Statistical analyses

Data were comprehensively collected from our reproductive center's case registration system, encompassing outpatient and inpatient medical records of patients, along with pregnancy outcome follow-ups. Analyses were conducted using SPSS statistical software (version 25.0; SPSS Inc.).

Given significant differences between fresh and frozen cycles, analyses were stratified accordingly. Neonatal outcomes were assessed via univariate analysis with stratification by cycle type, and group differences were compared using chi-square tests. Congenital anomalies were classified and described in detail based on ICD-10 codes, as detailed in eTable 1.

We incorporated baseline univariate findings and literature/consensus-based confounders into multivariate logistic regression models to address the retrospective cohort design and potential confounding effects on congenital anomalies. Variables analyzed included parental age subgroups, maternal BMI subgroups, infertility duration, diagnosis, cycle type (fresh/frozen), fertilization method, embryo stage, multiple pregnancies, embryo transfer number, infertility treatment, preterm delivery, and DYG exposure to identify independent risk factors for congenital anomalies.

Given the low proportion of missing maternal BMI data (under 4%), missing values were analyzed as a separate category without imputation. Multivariable logistic regression models, adjusted for the aforementioned confounders, were applied to all congenital anomaly categories. Additionally, stratified analyses by fresh/frozen cycles were conducted using the same covariates except for cycle type. Statistical significance was defined as a two-tailed *P*-value below 0.05.

## Results

### Neonatal outcomes with or without DYG exposure

**Fig. 1:**
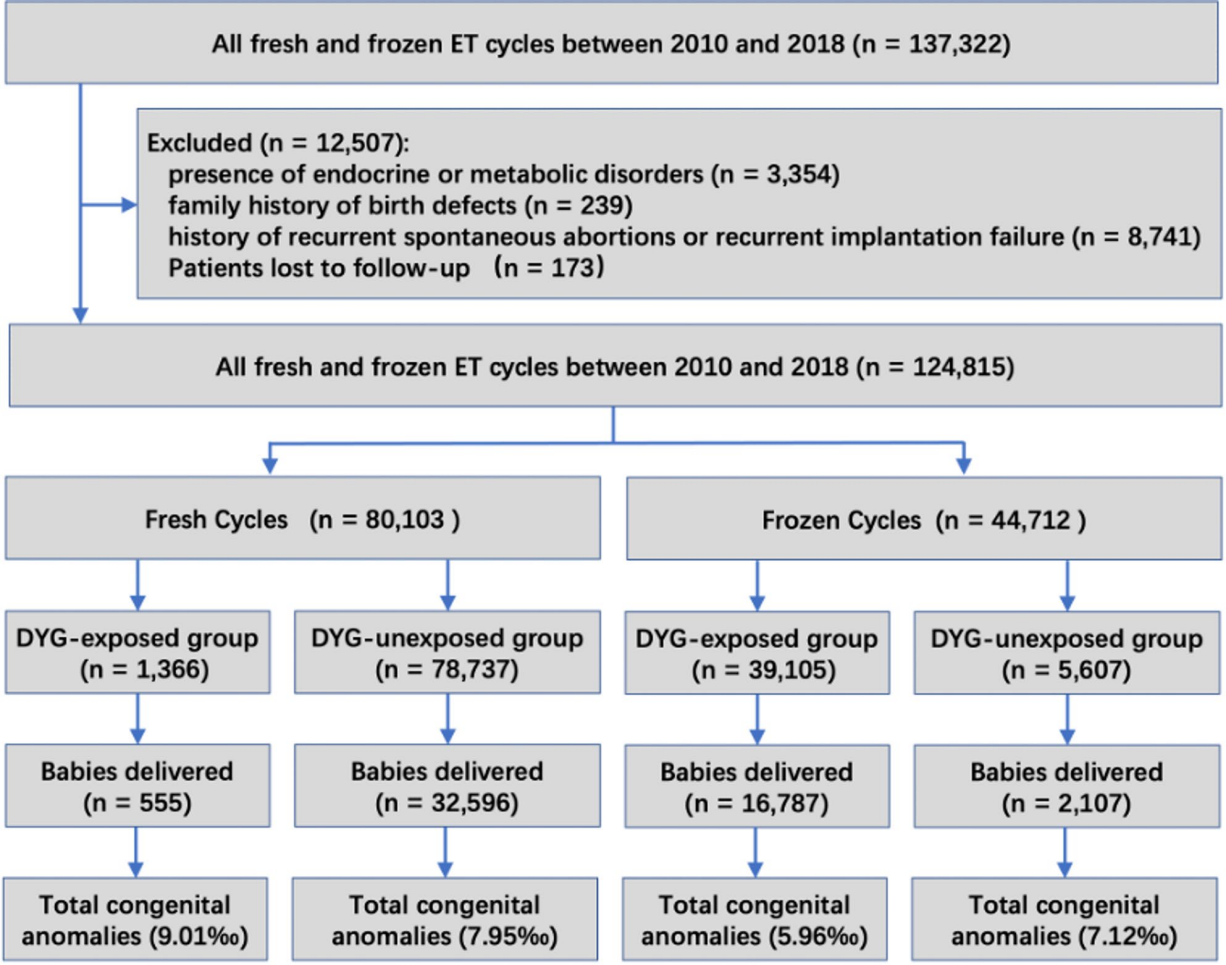
Flow diagram detailing the number of babies delivered included in the analysis

From 2010 to 2018, a total of 137,322 embryo transfer cycles were recorded. After applying exclusion criteria (detailed in Fig. 1), 124,815 cycles were analyzed, including 80,103 fresh cycles (64.2%) and 44,712 frozen cycles (35.8%), resulting in 52,175 live births. Baseline characteristics stratified by maternal DYG exposure are presented in eTable 2. Paternal age, gravidity, parity, and fertilization methods showed no significant differences between groups.

Neonatal outcomes are summarized in Table 2. No significant differences were observed between groups in stillbirth rates, live-born infant sex ratios, or fetal macrosomia proportions. However, among frozen embryo transfer (FET) cycles, neonates in the DYG-exposed group exhibited significantly higher birth weights and lower rates of low birth weight (LBW) and very low birth weight (VLBW) compared to the unexposed group.

**Table 2:** Neonatal outcomes of the study cohort according to the status of maternal DYG therapy

	Fresh cycles			Frozen cycles			
	DYG-exposed	DYG-unexposed	*P*-value	DYG-exposed	DYG-unexposed	*P*-value	
Babies delivered (n)	558	32,670		16,833	2,114		
Live newborns	555 (99.46%)	32,607 (99.81%)	0.100	16,788 (99.73%)	2,107 (99.67%)	0.597	
Stillbirths	3 (0.54%)	63 (0.19%)		45 (0.27%)	7 (0.33%)		
Sex of live newborns^a^							
Male (n)	278 (50.09%)	16,957 (52.00%)	0.366	8,953 (53.33%)	1,100 (52.21%)	0.340	
Female (n)	277 (49.91%)	15,638 (48.00%)		7,834 (46.67%)	1,006 (47.79%)		
Macrosomia (n)	28 (5.08%)	1,732 (5.37%)	0.767	1,273 (7.67%)	157 (7.57%)	0.873	
Low birth weight (n)	105 (19.06%)	5,983 (18.55%)	0.760	2,170 (13.07%)	397 (19.14%)	< 0.001	
Very low birth weight (n)	4 (0.73%)	403 (1.25%)	0.271	140 (0.84%)	34 (1.64%)	< 0.001	
Birth weight (g)	2,999.53 ± 619.22	2,992.90 ± 627.48	0.806	3,133.51 ± 617.51	3,034.34 ± 678.95	< 0.001	

Data are presented as mean ± standard deviation or percentage unless otherwise specified

*DYG* dydrogesterone

Macrosomia was defined as a birth weight of ≥ 4,000 g. Low birth weight was defined as a birth weight of < 2,500 g. Very low birth weight was defined as a birth weight of < 1,500 g

^a^There were 14 missing data about sex of live newborns

### Analysis of risks of congenital anomalies with DYG exposure

Congenital anomalies in the cohort are detailed in Table 3. The DYG-exposed and unexposed groups comprised 17,342 and 34,703 live births, respectively. Total congenital anomalies were significantly less frequent in the DYG-exposed group (105 cases, 6.05‰) versus the unexposed group (274 cases, 7.90‰; *P* = 0.020). Musculoskeletal malformations specifically showed lower incidence with DYG exposure (0.63‰ vs. 1.33‰; *P* = 0.025). While foot deformities differed significantly between groups (*P* = 0.004; eTable 3), other musculoskeletal subtypes like polydactyly demonstrated no statistical variation. No significant differences were observed in other anomaly categories.

**Table 3:** Congenital anomalies of the study cohort according to the method of maternal DYG therapy

	DYG-exposed		DYG-unexposed		*P*-value
	Count	Rate (‰)	Count	Rate (‰)	
Babies delivered (n)^a^	17,342		34,703		
Total congenital anomalies in baby delivered (n)	105	6.05	274	7.90	0.020
Congenital malformations of the nervous system	9	0.52	31	0.89	0.146
Congenital malformations of eye, ear, face, and neck	7	0.40	25	0.72	0.169
Congenital malformations of the circulatory system	37	2.13	83	2.39	0.563
Congenital malformations of the respiratory system	5	0.29	7	0.20	0.759
Cleft lip and cleft palate	13	0.75	17	0.49	0.245
Other congenital malformations of the digestive system	6	0.35	24	0.69	0.122
Congenital malformations of genital organs	8	0.46	9	0.26	0.229
Congenital malformations of the urinary system	6	0.35	16	0.46	0.547
Congenital malformations and deformations of the musculoskeletal system	11	0.63	46	1.33	0.025
Other congenital malformations	6	0.35	20	0.58	0.268
Chromosomal abnormalities, not elsewhere classified	3	0.17	9	0.26	0.760

*DYG* dydrogesterone

^a^Multi-malformation cases were divided into 11 malformation classifications above. There were 12 missing data about congenital anomalies in live newborn

Due to substantial differences in treatment protocols and pregnancy physiology between fresh and frozen cycles, we performed stratified analyses by cycle type (Table 4). In fresh cycles, no significant differences in congenital anomaly incidence were observed between DYG-exposed and unexposed groups, either before or after adjustment. In frozen cycles, an initial analysis suggested a significantly lower frequency of musculoskeletal anomalies in the DYG-exposed group both before (0.60‰ vs. 2.37‰; *P* = 0.020) and after adjustment for confounders (*P* = 0.009, OR 0.19, 95% CI 0.05–0.66). However, this isolated finding did not remain significant after rigorous correction for multiple testing using both the Bonferroni and False Discovery Rate (FDR) methods. No significant differences were observed in any other anomaly categories (detailed in eTable 1). Therefore, the stratified analysis confirms that DYG exposure is not associated with an altered risk of congenital anomalies in either fresh or frozen cycles.

**Table 4:** Congenital anomalies of the study cohort by maternal DYG therapy status

	**Fresh cycles**							**Frozen cycles**						
	DYG-exposed		DYG-unexposed		*P*-value	Adjusted^a^		DYG-exposed		DYG-unexposed		*P-*value	Adjusted^a^	
	Count	Rate (‰)	Count	Rate (‰)		*P*-value	Odds ratio (95% CI)	Count	Rate (‰)	Count	Rate (‰)		*P*-value	Odds ratio (95% CI)
**Babies delivered**^**b**^	555		32596					16787		2107				
Total congenital anomalies	5	9.01	259	7.95	0.633	0.923	0.96 (0.38, 2.39)	100	5.96	15	7.12	0.519	0.653	1.15 (0.62, 2.13)
Nervous system	0	0	31	0.95	> 0.999	0.993	-	9	0.54	0	0	0.610	0.987	-
Eye, ear, face and neck	1	1.80	25	0.77	0.355	0.390	2.42 (0.32, 18.19)	6	0.36	0	0	> 0.999	0.985	-
Circulatory system	2	3.60	79	2.42	0.394	0.914	0.92 (0.19, 4.34)	35	2.08	4	1.90	> 0.999	0.459	1.53 (0.50, 4.71)
Respiratory system	0	0	7	0.21	> 0.999	0.993	-	5	0.30	0	0	> 0.999	0.914	0.92 (0.19, 4.34)
Cleft lip and cleft palate	0	0	15	0.46	> 0.999	0.993	-	13	0.77	2	0.95	0.680	0.985	-
Digestive system	0	0	24	0.74	> 0.999	0.993	-	6	0.36	0	0	> 0.999	0.983	-
Genital organs	1	1.80	9	0.28	0.155	0.060	7.77 (0.92, 65.59)	7	0.42	0	0	> 0.999	0.983	-
Urinary system	0	0	14	0.43	> 0.999	0.993	-	6	0.36	2	0.95	0.222	0.351	0.42 (0.07, 2.58)
Musculoskeletal system	1	1.80	41	1.26	0.508	0.735	1.41 (0.19, 10.36)	10	0.60	5	2.37	0.020	0.009*	0.19 (0.05, 0.66)
Other congenital malformations	0	0	19	0.58	> 0.999	0.993	-	6	0.36	1	0.47	0.563	0.672	1.66 (0.16, 17.62)
Chromosomal abnormalities	0	0	8	0.25	> 0.999	0.993	-	3	0.18	1	0.47	0.377	0.984	-

*DYG* dydrogesterone

^a^Adjusted for: parental age, BMI, duration of infertility, frozen cycles, infertility diagnosis, treatment protocols, fertilization method, number of embryos transferred, embryo stage at transfer, multiple pregnancy, preterm delivery

^b^Multi-malformation cases were divided into 11 malformation classifications above. There were 12 missing data about congenital anomalies in live newborn

^*^It did not remain significant after rigorous correction for multiple testing with the application of both the Bonferroni and False Discovery Rate (FDR) methods

Multivariable logistic regression was conducted to identify factors associated with congenital anomalies (eTable 4). Variables analyzed included DYG exposure, parental age, maternal BMI, infertility duration/diagnosis, treatment protocols (fresh/frozen cycles), fertilization method, embryo stage, embryo transfer number, multiple pregnancies, and preterm delivery. All variables showing potential associations in univariate analyses were included. After adjusting for confounders, only preterm delivery remained independently associated with congenital anomalies, while DYG exposure showed no increased risk.

## Discussion

### Main findings

In this retrospective cohort study with a considerably large sample size of newborns, first-trimester DYG therapy did not increase the risk of congenital anomalies.

### Efficacy and satisfaction of DYG

DYG has been utilized since the 1960 s for managing threatened and recurrent miscarriages, with recent approval for LPS in assisted reproductive technology (ART) [20]. An estimated 113 million women and 20 million fetuses have been exposed to DYG across various indications [12]. Unlike progesterone, whose efficacy and fetal safety are well-established through decades of research, DYG’s applications have only recently been systematically investigated. Multiple randomized trials [6, 11–14], including the 2017 double-blind phase III Lotus I trial and 2018 open-label Lotus II trial, [12, 13] demonstrate comparable pregnancy outcomes between DYG and vaginal progesterone. Notably, a 2021 meta-analysis suggested higher pregnancy and live birth rates with oral DYG versus vaginal progesterone for LPS [21]. Furthermore, in a randomized study, DYG exhibits superior treatment tolerability and patient satisfaction compared to micronized progesterone [14].

### Offspring safety of DYG

Congenital anomaly is a critical public health issue and should be a research priority. While ART effectively addresses infertility, it was shown to be associated with an increased risk of congenital anomalies in some studies [22–27], while other studies demonstrated otherwise [28–30]. Notably, The underlying risk of congenital anomalies due to subfertility cannot be ignored [28]. Existing clinical trials [12, 13] comparing DYG and progesterone for LPS demonstrate comparable congenital anomaly rates, suggesting favorable benefit-risk profiles for both agents.

However, contradictory evidence exists: a case–control study [15] reported elevated congenital heart defect risks with DYG exposure (OR 2.71), though methodological limitations—such as inadequate adjustment for prior miscarriage, a known cardiac anomaly risk factor [19] —have drawn systematic critique [31–33]. Recent data further complicate this landscape. A Chinese prospective cohort [17] linked first-trimester DYG exposure to marginally higher birth defect rates (RR 1.13, 95% CI 1.06–1.21) versus progesterone (RR 1.09, 95% CI 0.97–1.13), though both risks remained statistically proximate. Intriguingly, chorionic gonadotropin—a physiologically elevated pregnancy hormone—exhibited higher teratogenic risk (RR 1.24, 95% CI 1.01–1.52) in this study, challenging assumptions about exogenous hormone causality.

Another study, using the WHO global safety database [18], showed an increased reporting of birth defects, mainly hypospadias and congenital heart defects, was found with DYG when compared to progesterone. In the study, although 3101 reports were related to the use of drugs for ART, there were only 145 with DYG and 1222 with progesterone. 374 birth defects were identified in those 3101 ART-related reports, with 60 cases linked to DYG versus 141 to progesterone. In contrast, our cohort of 52,175 IVF-conceived neonates were 33.3% DYG-exposed (17,342 cases). It documented 379 anomalies, including 105 DYG-associated and 274 progesterone-associated cases. While the WHO database maintains methodological rigor, its smaller DYG sample (145 cases) may limit generalizability. The observed discrepancies may stem from regional variations in luteal phase support protocols across countries/regions or incomplete drug inclusion in this database. Therefore, our findings provides robust real-world evidence regarding the teratogenic potential of DYG, offering insights into its association with neonatal malformations.

The congenital anomaly incidence among live-born infants was 7.26‰ in our study. A cross-comparison of congenital anomaly rates across studies with varying designs reveals heterogeneity. Our rate is consistent with international registries like the U.S. studies of ART-conceived infants (5.86 ‰) [23]. However, within China, reported rates vary widely. Official data from Beijing (2013–2015) reported rates ranging from 1.8‰ to 7.3‰ for fresh and frozen embryo transfer cycles [34], which are lower than our finding. Our observed rate is also lower than some national estimates (1.53%) and figures from certain ART populations (1.97%) [22], but aligns more closely with other retrospective studies, such as Zhu et al. (1.15% for IVF, 1.38% for ICSI) [35], which similarly captured defects apparent at or near birth. The most striking contrast is with the prospective cohort by Lv et al., which implemented intensive follow-up until 1 year of age and reported a cumulative prevalence of 13.9% [36]. This difference in rates reported across Chinese studies underscores the profound impact of surveillance methodology. The substantially higher rate in the latter study can be attributed to its prospective design, extended follow-up period, and systematic physical examinations, which significantly enhance the detection of late-manifesting and minor defects. The comparatively lower rate in our study can be attributed to several key design elements that this cross-comparison brings to the fore: the systematic exclusion of patients with established risk factors for birth defects [37, 38]; the restriction of analysis to live births, thereby excluding pregnancies terminated following prenatal diagnosis (detailed in eTable 5); and the absence of long-term, systematic postnatal follow-up, which limits the detection of minor or late-manifesting defects. Consequently, our estimates provide a conservative measure of immediately apparent anomalies.

We acknowledge the concern that, given the large number of subgroup comparisons and the very small event counts in most anomaly categories, the few nominally significant P-values in Tables 3 and 4 should be interpreted with caution. This is exemplified by the finding for musculoskeletal anomalies in frozen cycles, which was significant before correction (*P* = 0.009) but did not remain so after rigorous adjustment for multiple testing using both Bonferroni and False Discovery Rate methods. Consequently, these isolated findings are plausibly attributable to chance, and our primary conclusions are therefore anchored in the robust, overall malformation rate, which showed no increased risk associated with DYG. Future studies with larger cohorts and structured, long-term follow-up are warranted to obtain more stable and comprehensive data on specific birth defect subtypes following ART.

The long-standing debate regarding a potential link between progestins and nongenital malformations was comprehensively addressed and largely settled by Brent [39] in 2005. His work synthesized a substantial body of evidence demonstrating no biologically plausible mechanism or consistent epidemiological association between clinically used progestins and congenital anomalies such as limb reduction or congenital heart defects. Our findings on the safety of DYG are consistent with this well-established scientific consensus. The absence of an increased risk for malformations in our large cohort reinforces the conclusion that progestins, including DYG, are not teratogenic in this regard, thereby adding robust contemporary evidence to this resolved scientific issue.

### Strengths and limitations

The main strength of our study is its large sample size, with over 50,000 live-born infants. Additionally, stratification of progesterone-based luteal support into DYG-exposed and unexposed groups, combined with cycle-specific analyses (fresh vs. frozen), enabled clearer isolation of drug effects from confounding variables.

The limitations of our study are its retrospective nature and non-randomized design. Although dispensary information from our medical records was accurate, patients’ compliance with prescribed drugs was unknown. However, any nonadherence likely occurred equally between DYG-exposed and unexposed groups, minimizing bias in comparing progesterone formulations’ teratogenic risks. An other limitation is its reliance on congenital anomaly diagnoses at birth. Notably, certain major structural defects—particularly those manifesting symptomatically later in development—are frequently identified during postnatal assessments within the first year of life or beyond. Consequently, the lack of structured postnatal monitoring in our protocol may have led to underestimation of anomaly prevalence rates. Although longitudinal follow-up for a cohort exceeding 50,000 neonates presents significant challenges, we are committed to strengthening our follow-up protocols (e.g., 6-month and 12-month assessments) in future studies to obtain more precise data on congenital anomalies.

## Conclusion

After adjusting for potential confounding factors, DYG exposure was not associated with an increased incidence of congenital anomalies overall. These preliminary findings require further in-depth studies to be validated. Additionally, long-term monitoring of children's holistic health outcomes in exposed populations is recommended to assess delayed effects.

## Supplementary Information

Supplementary Material 1: Supplementary Table S1. Detailed Incidence of each classification of congenital anomalies of the study cohort according to the status of maternal DYG therapy. Supplementary Table S2. Baseline characteristics of the study cohort according to the method of maternal DYG therapy. Supplementary Table S3. Congenital anomalies of the musculoskeletal system according to the method of maternal DYG therapy. Supplementary Table S4. Multivariate logistic regression analysis for factors influencing the occurrence of congenital anomalies. Supplementary Table S5. Pregnancies terminated following prenatal diagnosis according to the method of maternal DYG therapy

## Data Availability

Data should be requested by email to the corresponding author (email: chihb@163.com) and must include the name and full contact information of the person and institution requesting the data, the research objectives, methodology, anticipated outcomes, plans for sharing results, and the purpose of requesting the data. Data requests under agreement will be subjected to appropriate confidentiality obligations and restrictions.

## Abbreviations

DYG: Dydrogesterone
LPD: Luteal phase defects
FDR: False Discovery Rate
IVF: In vitro fertilization
LH: Luteinizing hormone
LPS: Luteal phase support
IM: Intramuscular
ICSI: Intracytoplasmic sperm injection
FET: Frozen embryo transfer
LBW: Low birth weight
VLBW: Very low birth weight
ART: Assisted reproductive technology
